# Aneuploid CTC and CEC

**DOI:** 10.3390/diagnostics8020026

**Published:** 2018-04-18

**Authors:** Peter Ping Lin

**Affiliations:** Cytelligen, San Diego, CA 92121, USA; plin@cytelligen.com; Tel.: +1-858-336-5788; Fax: +1-858-509-9209

**Keywords:** iFISH, aneuploidy, circulating rare cells, liquid biopsy, molecular diagnostics, whole genome amplification (WGA) and next generation sequencing (NGS) of the single CTC, proteomic and genomic profiling of the single CRC, tumor protein p53 (*TP53*) and *CDKN2A* tumor suppressor gene mutations, programmed death-ligand 1 (PD-L1) and anaplastic lymphoma kinase (ALK)

## Abstract

Conventional circulating tumor cell (CTC) detection technologies are restricted to large tumor cells (> white blood cells (WBCs)), or those unique carcinoma cells with double positive expression of surface epithelial cell adhesion molecule (EpCAM) for isolation, and intracellular structural protein cytokeratins (CKs) for identification. With respect to detecting the full spectrum of highly heterogeneous circulating rare cells (CRCs), including CTCs and circulating endothelial cells (CECs), it is imperative to develop a strategy systematically coordinating all tri-elements of nucleic acids, biomarker proteins, and cellular morphology, to effectively enrich and comprehensively identify CRCs. Accordingly, a novel strategy integrating subtraction enrichment and immunostaining-fluorescence in situ hybridization (SE-iFISH), independent of cell size variation and free of hypotonic damage as well as anti-EpCAM perturbing, has been demonstrated to enable in situ phenotyping multi-protein expression, karyotyping chromosome aneuploidy, and detecting cytogenetic rearrangements of the *ALK* gene in non-hematologic CRCs. Symbolic non-synonymous single nucleotide variants (SNVs) of both the *TP53* gene (P33R) in each single aneuploid CTCs, and the cyclin-dependent kinase inhibitor 2A (*CDKN2A*) tumor suppressor gene in each examined aneuploid CECs, were identified for the first time across patients with diverse carcinomas. Comprehensive co-detecting observable aneuploid CTCs and CECs by SE-iFISH, along with applicable genomic and/or proteomic single cell molecular profiling, are anticipated to facilitate elucidating how those disparate categories of aneuploid CTCs and CECs cross-talk and functionally interplay with tumor angiogenesis, therapeutic drug resistance, tumor progression, and cancer metastasis.

## 1. Background

Circulating tumor cells (CTCs) are cancer cells shed from primary or metastatic solid tumors into peripheral blood, whereas circulating endothelial cells (CECs) are derived from endothelial cells (ECs) of blood vessels into circulation. Clinical relevance of CTCs in tumor metastasis and prognosis [1,2,3], CECs in tumor angiogenesis [4], and CEC clusters in carcinoma [5] have been substantially discussed elsewhere. 

Aneuploidy is the hallmark of malignant cells [6,7]. In addition to aneuploid cancer cells and aneuploid endothelial cells localized in tumor tissues, existence of aneuploid CTCs and CECs in peripheral blood has been recently reported [8]. Nonetheless, how those diverse types of aneuploid malignant cells cross-talk and inter-play in tumor formation and metastasis remains to be further investigated. 

Detection of circulating rare cells (CRCs), including both CTCs and CECs, is the most representative of liquid biopsy due to its unique availability of frequent and non-invasive detecting tumor cells in carcinoma patients. However, the expanded application of the majority of the current CTC detection technologies, relying on either cell size or positive expression of particular anchor proteins on cancer cell surface is significantly limited. 

In the present short review, conventional CTC detection strategies, and a novel integrated SE-iFISH platform, applied to examine cytogenetic gene rearrangements in cancer cells, and to co-detect, characterize, molecularly profile aneuploid CTCs as well as CECs, are discussed.

## 2. Aneuploidy

Aneuploidy refers to the abnormal alternation (either gain or loss) of chromosomes in a cell. Unlike “constitutional aneuploidy”, which is derived from inappropriate chromosome segregation in meiosis of germ cell formation and present throughout whole organisms, the “somatic whole-chromosome or large segmental aneuploidy” is from deviation in mitosis, and only some of cells are affected [9]. Somatic aneuploidy is the most common characteristic of human carcinomas [6,7]. Approximately 90% of solid tumors and 75% of hematological carcinomas exhibit aneuploidy [10,11]. In particular, aneuploid chromosome 8 (Chr 8) was observed in neoplasm cells of several solid tumors, including lung, gastric, pancreatic, colon, bladder, esophageal, and hepatocellular carcinomas, etc. [12]. Besides, aneuploid Chr 8 was also revealed in endothelial cells of tumor mass, known as tumor ECs [13,14].

Aneuploidy is a cellular transformation-related dynamic chromosome mutation event regulated by a number of mitotic genes. Mutations of those mitotic genes were identified in cancer cells, implicating such mutation in induction of mis-chromosome segregated aneuploidy in neoplasm cells [15]. It has been recognized that aneuploidy drives cancer development and evolution [16].

Aberrant ploidy of extra-chromosome in cancer cells was found to lead to genomic instability [17]. In addition, chromosome or regional aneuploidy has been realized to significantly impact expression of hundreds of genes that are either gained or lost in carcinoma cells. Moreover, aneuploidy per se affects transcription of multiple genes due to activation or inhibition of several intracellular signal transduction pathways in response to chromosome copy number variation [18], resulting in a profound variety of phenotypes, which further contributes to tumor heterogeneity, drug resistance, and therapy failure [16]. 

Given the unique property of aneuploidy in neoplasm cells, novel anti-cancer strategies targeting aneuploidy for cancer therapy have recently been successfully developed [19,20]. 

## 3. CTC and CEC

CTCs and CECs constitute the principal entity of non-hematologic circulating rare cells in circulation. 

CTCs in blood and disseminated tumor cells (DTCs) in bone marrow and lymph nodes, are precursors and surrogate markers of tumor metastasis and relapse [1,3,21]. Superior to routine clinic diagnostics, quantitative and qualitative detection and characterization of CTCs/DTCs have been utilized to evaluate therapeutic efficacy [22,23], monitor postsurgical cancer relapse [24,25] and drug resistance in both carcinoma patients [23,26,27] as well as metastatic “patient derived xenograft” (mPDX) tumor animal models [28]. The eminent advantage of quantified CTCs correlating with prostate cancer patients’ prognosis in 5 of randomized phase III clinical trials has recently been demonstrated and reported [2].

CECs are relevant to the process of angiogenesis. Despite their pivotal roles in cardiovascular diseases [29], CECs are the biomarker of neoplasm [4,30], and play an integral part in neovascularization, known as tumor angiogenesis, which is essential for invasive tumor growth and metastasis [31]. An increased number of viable CECs correlates with plasma concentration of vascular endothelial growth factor (VEGF) [32], tumor progression, and therapeutic response [30,33,34]. Though there are several subsets of CECs, CD31 is the most common molecule shared by all diverse subtypes [35].

## 4. Conventional Strategies and Current Progress in CTC Detection

The majority of the conventional strategies to isolate CRCs (including CTCs and CECs) rely on positively expressed specific cell surface anchor molecules. Application of such technologies is significantly complicated due to constant or dynamic heterogeneity of the anchor proteins. Regarding CTC detection, the majority of the current methodologies are restricted to the specific subset of CTCs showing positive expression of EpCAM [36], whereas absence of EpCAM was reported on as many as 30% of the examined 134 epithelial solid tumors [37]. Moreover, inherit dynamic expression [38,39], highly heterogeneous [12,40], or down-regulation of the anchor protein EpCAM which associates with epithelial-to-mesenchymal transition (EMT) and cancer progression as well as metastasis [41,42], will result in failure to isolate those “uncapturable” EpCAM negative CTCs by means of the anti-EpCAM strategy or its derived techniques [43,44]. 

Another approach to isolate CTCs is the cell size based filtration, designed to enrich CTCs via filtering out white blood cells (WBCs, 5 μm) [45]. Considering that a tremendous quantity of primary CTCs in cancer patients are of small cell size (≤ WBCs), 70% of the hepatocellular carcinoma (HCC) CTCs detected in either pre- or post-surgery patients are small CTCs [25], the obvious drawback of losing most of the clinically relevant small CTCs [25,46] for such a cell filtration strategy is unneglectable [47,48]. 

**Figure 1:**
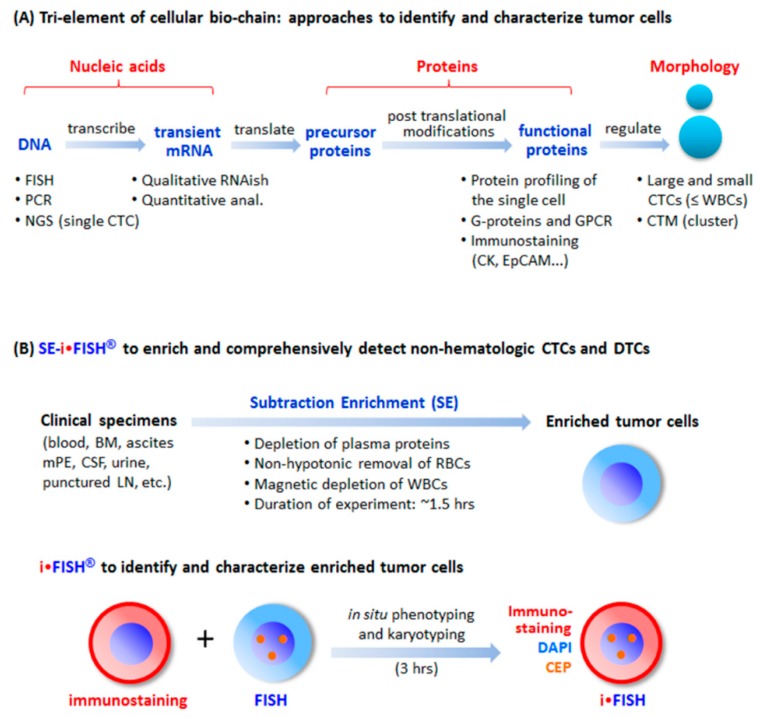
Tri-element of the cellular bio-chain and approaches to detect circulating tumor cells (CTCs). (**A**) Conventional strategies to detect and characterize CTCs. A cellular bio-chain consists of triple elements of nucleic acids, proteins, and cell morphology. Conventional approaches for CTC detection and characterization, respectively addressing each element, are indicated. (**B**) Schematic depiction of the principle of SE-iFISH. Subtraction Enrichment (SE): Clinical specimens including blood, bone marrow (BM), ascites, malignant pleural effusion (mPE), cerebrospinal fluid (CSF), urine, and punctured lymph node (LN) are processed to remove both WBCs by means of immunomagnetic beads, and RBCs via Cytelligen’s non-hematologic cell separation matrix based centrifugation, to enrich non-hematologic CRCs including large, small or the clusters of CTCs and CECs. iFISH: iFISH coordinates all tri-elements along the cellular bio-chain. In situ phenotypic immunostaining of multiple biomarker proteins and karyotypic FISH carried out using a centromere probe (CEP) are simultaneously co-performed on the identical enriched target cell.

Currently, the most common strategy applied for CTC identification is to immunostain intracellular fibrous protein cytokeratin (CK). Similar to EpCAM, CK is down-regulated during EMT [39,49], which inevitably results in false negative detection of such “invisible” CTCs. Summarized in Figure 1A, additional efforts respectively addressing each individual element of the cellular bio-chain, including digital RT-PCR [50], single or pooled CTCs next generation sequencing (NGS) analysis [51,52], FISH [53,54], quantitative [55] and qualitative (RNAish) [56] analyses of mRNA in transient, CTC protein profiling performed by the single cell proteomics [57], G-proteins and G-protein coupled receptor (GPCR) [55,58,59], etc., were made in attempts to improve identification and characterization of tumor cells. Nonetheless, tri-element methods, rather than single element method, should be integrated into a comprehensive strategy to obtain fundamental improvement for effective detection of highly heterogeneous CTCs.

## 5. Comprehensive in Situ Phenotypic, Karyotypic, and Cytogenetic characterization as well as Classification of CTCs by SE-iFISH

An effective CTC detection strategy is constituted by both efficient isolation and adequate identification. Published efforts to date have only tried to respectively improve either isolation or identification, but rarely both [12]. It is therefore imperative to develop a comprehensive strategy to effectively isolate, identify, characterize, and classify the full spectrum of highly heterogeneous CTCs.

Accordingly, aside from respectively addressing nucleic acids, proteins, or cell morphology alone, an integrated subtraction enrichment (SE), and immunostaining-FISH (iFISH) which coordinates all three elements of nucleic acids, proteins, and cell morphology along the cellular bio-chain (Figure 1A) has been systematically developed to efficiently isolate and effectively identify CTCs [8,12,40,60,61]. Explicit description of the updated SE-iFISH experimental process in detail has been published [8]. Schematically depicted in Figure 1B, non-hematologic rare cells, including CTCs in various types of specimens, are enriched following non-hemolytic removal of RBCs and maximum depletion of most WBCs (4-5 logs). Since the prototyping SE applied for lung cancer CTC study was initially reported in 2009 [62], significant improvement was made to efficiently enrich various CTCs, DTCs, and circulating tumor microemboli (CTM) in different types of cancer patients or PDX tumor mouse models [28], despite heterogeneous expression of surface anchor molecule(s) on CTCs or cell size variation. Rapidly enriched viable tumor cells, unperturbed by antibody resistance and free of hypotonic damage, are eligible for primary tumor cell culture and a series of downstream analyses. Recently, epidermal growth factor receptor-tyrosine kinase inhibitors (EGFR-TKIs) target therapy (Gefitinib/Irresa^®^) oriented examination of EGFR mutations, performed on the enriched single non-small cell lung cancer CTCs [63], and NGS-guided in vitro drug screening carried out on the cultured metastatic breast cancer cells enriched from patient’s cerebrospinal fluid by SE, to successfully select the chemotherapeutic agent palbociclib (the synthetic CDK4/6 inhibitor) upon the identification of a single nucleotide variant (SNV), have been reported [64].

The conventional FISH approach was previously applied by others in an attempt to improve the identification of CTCs [53,54]. However, such circumscribed efforts were complicated due to complicacy of hematologic compositions of blood. Substantial improvement of such attempts is required for better performance with respect to in situ co-detection of chromosome aneuploidy and multiple tumor biomarker expression on or in CTCs. 

**Figure 2:**
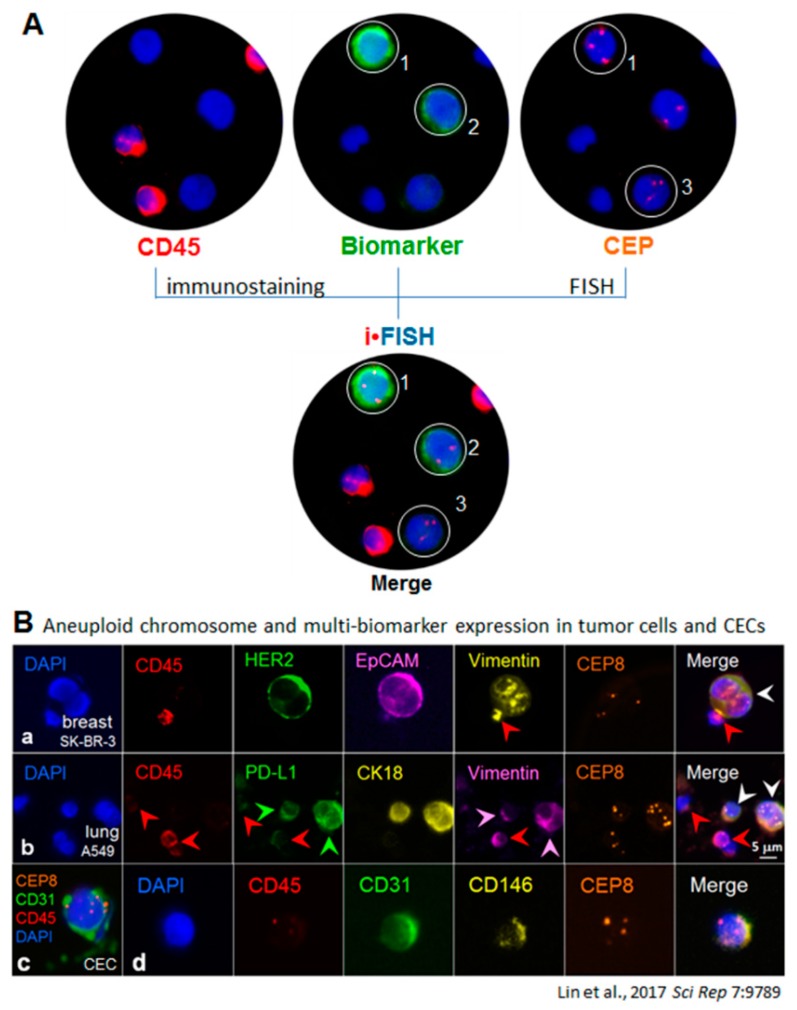
In situ phenotypic and karyotypic identification as well as comprehensive characterization of tumor cells by iFISH. (**A**) Principle of iFISH depicted with cellular images. in situ phenotypic immunostaining and karyotypic FISH demonstrates that among three of the enriched non-hematologic cells (CD45^−^), Cells 1 and 2 are positive for tumor biomarker immunostaining, whereas Cells 1 and 3 have aneuploid chromosomes revealed by FISH performed with the Vysis Centromere Probe (CEP8 in this image) (Abbott Laboratories, USA). However, merged iFISH image indicates that Cells 1–3 are tumor cells, showing that Cell 1 has triploid chromosome (Chr 8 in this study) with strong tumor biomarker expression (CK18 in this case); Cell 2 possesses disomy 8 with low CK18 expression; and Cell 3 has trisomy 8 with absence of CK18 [12]. (**B**) Multi-tumor biomarker-iFISH (6-fluorescence color). Displayed images of iFISHed carcinoma cells are scanned, identified, located, acquired, and analyzed by the Metafer-iFISH^®^ automated CRC image scanning and analyzing system [8]. **B-a**, HER2/EpCAM/Vimentin-iFISH reveals phenotypic expression of CD45 (red), HER2 (green), EpCAM (pink), and Vimentin (yellow) on either diploid WBC (red arrow, CD45^+^/Vimentin^+^) or the enriched breast cancer cells (white arrow, CD45^−^/HER2^+^/EpCAM^+^/Vimentin^+^) with chromosome abnormalities of Chr 8 deletion (monoploid Chr 8, orange). **B-b**, 2 of the enriched aneuploid lung cancer cells (white arrows) are CD45^−^/PD-L1^+^ (green arrows)/CK18^+^ (yellow)/Vimentin^+^ (pink arrows). Residual WBCs (red arrows) are CD45^+^/PD-L1^+^/CK18^−^, and one of WBCs shows high expression of vimentin (red arrow). **B-c**, a non-hematologic CD45^−^/CD31^+^ CEC with aneuploid Chr 8 enriched from a breast cancer patient is revealed. **B-d**, some of endogenous aneuploid CECs co-express CD146, showing CD45^−^/CD31^+^/CD146^+^ with trisomy 8 in this particular cell [8].

Given the existing technical hurdles, and in view of the unique and extraordinary significance in terms of performing in situ phenotyping of multiple tumor biomarkers’ protein expression and karyotyping chromosome aneuploidy in CTCs, a novel immunostaining-FISH (i•FISH^®^, Cytelligen, San Diego, CA, USA) has been developed to comprehensively identify and characterize non-hematologic aneuploid tumor cells (Figure 1B and Figure 2) [12]. Examined biomarker proteins are not restricted to the cell surface, but may also exist in the cytoplasm or nucleus [60,65]. Since i•FISH^®^ technology was reported for the first time on gastric CTC study [23], stepwise substantial improvement has been systematically built up to yield maximum efficiency and optimized flexibility for expeditious in situ co-detection of multiple tumor biomarkers or relevant proteins (such as PD-L1, CK, EpCAM, Vimentin, human epidermal growth factor receptor 2 (HER2), CD44, CD133, PSMA, GFAP, CD31, etc.), and aneuploidy of chromosome in CTCs at once [8,28,40,64]. “iFISHed” CTCs are classified into diverse subtypes by their identified tumor biomarker(s) and chromosome ploidy. It has been reported that different subpopulations of CTCs correlate with cancer recurrence in hepatocellular carcinoma patients [25], or chemotherapeutic resistance to cisplatin in both gastric cancer patients and PDX mice [23,28]. 

**Figure 3:**
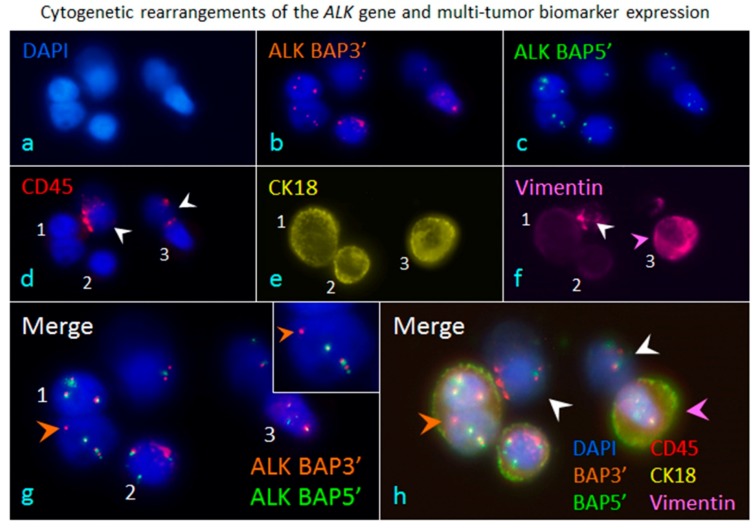
iFISH to co-detect cytogenetic rearrangements of the *ALK* gene and multi-tumor biomarker expression in cancer cells enriched from blood. A blood specimen containing adenocarcinomic NSCLC cells A549 was subjected to subtraction enrichment, followed by ALK/CK18/Vimentin-iFISH to quantitatively and qualitatively co-detect Chr 2 cytogenetic rearrangements of the broken apart *ALK* gene, and expression of CK18 as well as the mesenchymal marker Vimentin in enriched cells (**a**). The iFISH was performed using Vysis 3′ (orange, (**b**)) and 5′ (green, (**c**)) ALK Break Apart Probes (BAP) (Abbott Laboratories, Abbott Park, IL, USA). In situ immunofluorescence staining shows that two of cells are WBCs (CD45^+^, white arrows, (**d**)), and the rest of the enriched cells are the spiked non-hematologic cancer cells coded 1–3 (CD45^−^). Cell 1 has two nuclei. All the enriched cancer cells have positive expression of CK18 (CK18^+^/CD45^−^, (**e**)), whereas only cancer cell 3 has high amount of mesenchymal marker Vimentin co-expressed (Vimentin^+^/CK18^+^/CD45^−^, pink arrow, (**f**)), Cells 2 and 3 do not express Vimentin. One of the WBCs reveals significant positive staining of Vimentin (Vimentin^+^/CD45^+^, white arrow, (**f**)). Analysis of ALK-iFISH detection ((**g**) of merged (**b**,**c**)) shows a positive signal of the broken apart *ALK* gene (the single isolated orange dot of BAP3′ FISH signal, orange arrow) in the lung cancer cell-1(CK18^+^/Vimentin^−^/CD45^−^). An enlarged image of the positive break apart signal of ALK-iFISH is illustrated in top-right corner. The intact *ALK* genes are displayed by either adjacent (green and orange) or overlapped (yellow) ALK BAP3′ and ALK BAP5′ FISH signals (solid or diffused dots) in tumor cells 1–3 and WBCs. (**h**) reveals the overlapping image of merged 6 fluorescence channels (**a**–**f**), showing a CK18^+^/Vimentin^−^ cancer cell possessing the incomplete *ALK* gene (orange arrow), the CK18^+^/Vimentin^+^ double positive cancer cell (pink arrow) and WBCs (white arrows). All the images were acquired and analyzed by the Metafer-iFISH^®^ automated CRC scanning and analysis system (Zeiss, MetaSystems, and Cytelligen).

In addition to karyotypically examining aneuploidy of chromosomes in cancer cells, iFISH is also uniquely capable of qualitatively and quantitatively co-detecting multiple tumor biomarkers’ expression and cytogenetic gene rearrangement, such as anaplastic lymphoma kinase (*ALK*) gene [66] in adenocarcinomic non-small cell lung cancer (NSCLC) cells, as shown in Figure 3 (ALK-iFISH, Cytelligen, San Diego, CA, USA). 

Primary cancer cells in carcinoma patients are extremely heterogeneous. Evolutionary instability or variation of primary neoplasm cells, in terms of cytogenetic and/or phenotypic conversion between positivity and negativity following tumor progression or therapeutic treatments, is highly and constantly dynamic. Unlike conventional pathological biopsy routinely performed once to detect positivity of the therapeutic target (e.g., *ALK*, HER2, EGFR, etc.) on primary or metastatic lesions, continuously cytogenetic and/or phenotypic monitoring of dynamic target status on circulating carcinoma cells, such as the described *ALK* gene rearrangements, phenotypic protein expression of HER2, stem cell markers (CD133, CD44v6, EpCAM), immunotherapeutic marker PD-L1, and mesenchymal marker vimentin, etc. (Figure 2B) [8], provides a unique, reliable, and convenient approach for more appropriate and accurate evaluation and selection of the expanded scope of cancer patients suitable for the targeted therapy. 

## 6. Co-Detection of Aneuploid CTCs and CECs

CTCs with cytogenetic abnormalities of chromosome aneuploidy (such as aneuploid Chr 8) were previously reported [23,53,54,67]. 

**Figure 4:**
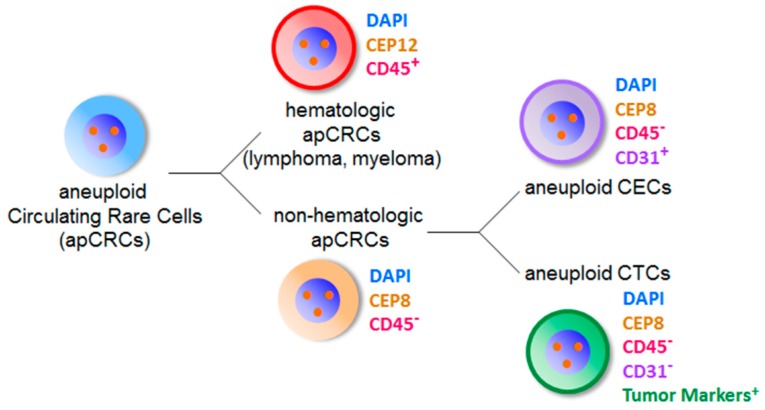
Classification of aneuploid circulating rare cells identified by iFISH. Aneuploid circulating rare cells (apCRCs in blue color) identified by iFISH are classified into two diverse categories of hematologic and non-hematologic apCRCs. All the hematologic apCRCs have positive CD45 staining (red color, CD45^+^) on the cell surface. The most representative hematologic apCRCs with cytogenetic abnormalities of Chr 12 are tumor cells of lymphoma and multiple myeloma. None of the non-hematologic apCRCs (orange color) have CD45 expressed on the cell surface (CD45^−^). The majority of non-hematologic apCRCs with aneuploid Chr 8 is composed of aneuploid CECs showing positive CD31 expression on the cell surface (pink color, CD31^+^), and aneuploid CTCs which do not express CD31 (CD31^−^) but tumor biomarker(s) (green color).

“Tumor endothelial cells” located on blood vessel of tumor tissues are a specific population of CD31^+^ ECs with aneuploid Chr 8 [14,68]. It is reasonable to speculate that aneuploid ECs fall off from blood vessel into circulation and subsequently turn into CD31^+^ aneuploid tumor CECs. Depicted in Figure 2B-c/d, existence of such tumor CECs with aneuploidy of Chr 8 in peripheral blood has been recently demonstrated by SE-iFISH [8]. Illustrated in Figure 4, both hematologic and non-hematologic aneuploid CRCs, identified by iFISH, constitute the primary entity of aneuploid CRCs (apCRCs). The most representative populations of hematologic apCRCs (aneuploid Chr 12) are tumor cells of lymphoma and myeloma. Non-hematologic apCRCs with aneuploid Chr 8 are composed of aneuploid CTCs and aneuploid CECs. Effective distinguishing of aneuploid CTCs vs. aneuploid CECs ensures high specificity with respect to detecting CTCs performed by iFISH. 

## 7. Proteomic and Genomic Profiling of Single Aneuploid CTCs and CECs 

Adequate approaches for molecular profiling, including both protein and genomic profiling performed on single CTC have been established. Protein profiling performed by single cell proteomics on the targeted individual CTC is expected to complement transcriptomic and genomic characterization [57]. Concerning cellular genotyping, a variety of strategies were proven applicable to carry out genomic profiling of single CTCs [51,52]. 

Gene mutations may lead to the inactivation of functional proteins they encode. By means of a non-laser microscopic single cell manipulator (NMSCM, Cytelligen) to collect intact individual iFISHed CRCs detected in various types of carcinoma patients [8], non-synonymous SNVs and additional gene mutations were identified for the first time by us, in the collected single aneuploid CTCs and CECs following whole genome amplification (WGA) of each of the collected cells. Preliminary NGS analyses performed on the single aneuploid CTCs and CECs detected by SE-iFISH in a population of lung, breast, and renal cell carcinoma patients indicated that, in contrast to the relatively low mutation frequency of *TP53* in primary carcinoma lesions, a high mutation frequency of tumor suppressor gene *TP53*, encoding the tumor suppressor protein p53 [69], was shown in every aneuploid CTC across all the cancer patients subjected to SE-iFISH. Specific *TP53* gene mutation results were pinpointed in non-functional phenotypic variants (P33R) of the tumor suppressor p53 in the detected circulating neoplasm cells.

An additional distinct, non-synonymous SNV identified in another tumor suppressor gene, *CDKN2A*, was found to be shared by most of the examined single aneuploid CECs in all the inspected subjects of the same population of patients with diverse carcinomas (our unpublished, ongoing study). *CDKN2A* encodes two different tumor suppressor proteins: p14, the alternate reading frame protein (ARF) to protect p53 from being broken down; and p16, known as the “inhibitor of CDK4” (INK4a), which is the inhibitor of intracellular endogenous CDK4/6. p16 activates retinoblastoma (Rb) family of proteins, and subsequently blocks traversal from G1 to S-phase [70]. Such regulatory effect is speculated to be impaired when the tumor suppressor gene *CDKN2A* is mutated in aneuploid CECs. Existence of disparate gene mutations in aneuploid CTCs and CECs, respectively, indicates striking diversity between these two categories of aneuploid circulating cells. 

The clinical relevance of aneuploid CECs has remained unknown since their existence was reported [8]. Given that triploid gastric CTCs are resistant to the chemotherapeutic agent cisplatin [23], and moreover, aneuploid tumor ECs possess resistance to both anti-angiogenic drugs [71] and the anti-cancer therapeutic agent, vincristine [72], it is anticipated that effective detection of aneuploid CECs derived from aneuploid ECs may promote more precise evaluation of therapeutic efficacy and tumor progression in carcinoma patients.

## 8. Conclusions

There are 22 worldwide ongoing multi-center CTC clinical trials, involving 20 biomarkers for CTCs and 14 for DTCs [3]. The established SE-iFISH strategy, facilitated by the newly developed Metafer-iFISH^®^ automated CRC image scanning and analysis system (Carl Zeiss, Oberkochen, Germany; MetaSystems, Altlussheim, Germany; and Cytelligen) [8], provides a unique comprehensive platform to effectively detect, phenotypically and karyotypically characterize CRCs with cytogenetic abnormalities of chromosome aneuploidy or gene rearrangements (such as *ALK* gene) in situ, in both patients and PDX tumor animal models. Diverse subtypes of CRCs, classified by biomarker expression and chromosome ploidy, are relevant to distinct clinical utilities. 

A series of intriguing questions, including whether identified mutant *TP53* and *CDKN2A* tumor suppressor genes, respectively shared by CTCs and CECs, could function as a driver to promote those cells into circulation, whether aneuploid CECs in motion could provide a microenvironment for CTCs to facilitate metastatic lesion formation, and how aneuploid CTCs and CECs correlate with tumor formation and progression remains unclear. Extensive co-investigation of aneuploid CTCs and CECs will shed light on how the diverse categories of aneuploid cells in circulation cross-talk and functionally interplay with tumor angiogenesis, progression, and metastasis.
